# Whole Genome Sequencing for Surveillance of Antimicrobial Resistance in *Actinobacillus pleuropneumoniae*

**DOI:** 10.3389/fmicb.2017.00311

**Published:** 2017-03-06

**Authors:** Janine T. Bossé, Yanwen Li, Jon Rogers, Roberto Fernandez Crespo, Yinghui Li, Roy R. Chaudhuri, Matthew T. G. Holden, Duncan J. Maskell, Alexander W. Tucker, Brendan W. Wren, Andrew N. Rycroft, Paul R. Langford

**Affiliations:** ^1^Section of Paediatrics, Department of Medicine, Imperial College LondonLondon, UK; ^2^Animal and Plant Health AgencyBury St Edmunds, UK; ^3^Department of Veterinary Medicine, University of CambridgeCambridge, UK; ^4^The Wellcome Trust Sanger InstituteCambridge, UK; ^5^Faculty of Infectious and Tropical Diseases, London School of Hygiene and Tropical MedicineLondon, UK; ^6^Department of Pathology and Pathogen Biology, The Royal Veterinary CollegeHatfield, UK

**Keywords:** animal infections, antimicrobial resistance genes, integrative conjugative elements, plasmids, genomics, respiratory tract, *Pasteurellaceae*

## Abstract

The aim of this study was to evaluate the correlation between antimicrobial resistance (AMR) profiles of 96 clinical isolates of *Actinobacillus pleuropneumoniae*, an important porcine respiratory pathogen, and the identification of AMR genes in whole genome sequence (wgs) data. Susceptibility of the isolates to nine antimicrobial agents (ampicillin, enrofloxacin, erythromycin, florfenicol, sulfisoxazole, tetracycline, tilmicosin, trimethoprim, and tylosin) was determined by agar dilution susceptibility test. Except for the macrolides tested, elevated MICs were highly correlated to the presence of AMR genes identified in wgs data using ResFinder or BLASTn. Of the isolates tested, 57% were resistant to tetracycline [MIC ≥ 4 mg/L; 94.8% with either *tet*(B) or *tet*(H)]; 48% to sulfisoxazole (MIC ≥ 256 mg/L or DD = 6; 100% with *sul2*), 20% to ampicillin (MIC ≥ 4 mg/L; 100% with *bla*_ROB-1_), 17% to trimethoprim (MIC ≥ 32 mg/L; 100% with *dfrA14*), and 6% to enrofloxacin (MIC ≥ 0.25 mg/L; 100% with GyrAS83F). Only 33% of the isolates did not have detectable AMR genes, and were sensitive by MICs for the antimicrobial agents tested. Although 23 isolates had MIC ≥ 32 mg/L for tylosin, all isolates had MIC ≤ 16 mg/L for both erythromycin and tilmicosin, and no macrolide resistance genes or known point mutations were detected. Other than the GyrAS83F mutation, the AMR genes detected were mapped to potential plasmids. In addition to presence on plasmid(s), the *tet*(B) gene was also found chromosomally either as part of a 56 kb integrative conjugative element (ICE*Apl1*) in 21, or as part of a Tn*7* insertion in 15 isolates. Our results indicate that, with the exception of macrolides, wgs data can be used to accurately predict resistance of *A. pleuropneumoniae* to the tested antimicrobial agents and provides added value for routine surveillance.

## Introduction

Antimicrobial resistance (AMR) in bacteria from food-producing animals is a growing concern (Michael et al., 2015). Extensive use of antimicrobial agents for treatment and prevention of diseases fosters an environment in which resistance determinants are acquired and maintained by pathogens, as well as commensal bacteria. In the UK, the swine industry accounts for a large proportion of the antimicrobial agents sold for use in food-producing animals; with tetracyclines, beta-lactams, and trimethoprim/sulphonamides being the most commonly used antimicrobial agents (Burch, 2005; Borriello, 2013).

*Actinobacillus pleuropneumoniae* is a major contributor to swine respiratory disease, causing considerable economic losses worldwide. Strategies to reduce the incidence and severity of disease include good husbandry, vaccination, and antibiotic treatment. The latter is essential to limit the severity and spread of pleuropneumonia. Knowledge of resistance profiles for *A. pleuropneumoniae* is required to inform treatment decisions. Furthermore, this information contributes to the larger picture of AMR in bacteria of animal origin (Hendriksen et al., 2008; El Garch et al., 2016).

Typically, determination of antimicrobial susceptibility is done either by disk diffusion or minimum inhibitory concentration (MIC) assays. Identification of the genetic determinants of resistance not only corroborates phenotypic results, but is also useful for epidemiological purposes, as there are often multiple different genes that can confer resistance to a given antimicrobial agent. Both PCR and microarray hybridization have been used to detect the presence of genes encoding resistance phenotypes, but these assays are limited to detecting the sequences tested, and do not allow direct detection of mutations conferring resistances (Frye et al., 2010; Ledeboer and Hodinka, 2011). It has recently been proposed that whole genome sequencing (wgs) may be an alternative for routine surveillance of resistance profiles and for identification of emerging resistances (Zankari et al., 2013). We recently used wgs to identify (for the first time) *dfrA14* as the genetic determinant of trimethoprim resistance detected in 16 clinical isolates of *A. pleuropneumoniae* (Bossé et al., 2015b). In this study, we compare the MIC profiles for nine antimicrobial agents with detection of resistance genes in wgs data of 96 isolates.

## Materials and Methods

### Bacterial Strains and Antimicrobial Susceptibility Testing

A total of 96 clinical *A. pleuropneumoniae* isolates from the UK, previously tested for trimethoprim resistance (Bossé et al., 2015b), were analyzed using the agar dilution susceptibility assay, according to the CLSI VET01-A4 guidance (Clinical and Laboratory Standards Institute [CLSI], 2013), for determination of MICs for tetracycline, ampicillin, sulfisoxazole, enrofloxacin, erythromycin, tilmicosin, tylosin, and florfenicol. Some samples were re-tested for resistance using the disk diffusion susceptibility test according to the CLSI VET01-A4 guidance (Clinical and Laboratory Standards Institute [CLSI], 2013). *A. pleuropneumoniae* ATCC 27090 and *Histophilus somni* ATCC 70025 were used as controls for all susceptibility tests. All clinical *A. pleuropneumoniae* isolates had been cultured from pneumonic lungs of pigs submitted for post-mortem to the then Animal Health and Veterinary Laboratory Agency (now Animal and Plant Health Agency) diagnostic laboratories in England (see Supplementary Table S1 for details). The serovar of each isolate was determined by PCR as previously described (Bossé et al., 2014).

### Genome Sequencing and Analysis

Genomic DNA was prepared from all isolates using the FastDNA Spin kit (MP Biomedicals), and 0.5 μg were used for library preparation and paired-end sequencing (Illumina HiSeq 2000), as previously described (Howell et al., 2013; Bossé et al., 2015b; Weinert et al., 2015). Cutadapt (Martin, 2011) was used to remove Illumina adapter sequences, and Sickle^1^ was used to trim low-quality sequences from the ends of reads, prior to assembly into contigs using Velvet 1.2.08 (Zerbino and Birney, 2008) and VelvetOptimiser 2.2.5 (Gladman and Seemann, 2012). Assemblies with N50 < 10000 were excluded from further analysis. The draft genome sequence for each isolate in this study has been deposited in the European Nucleotide Archive^2^, and accession numbers are listed in Supplementary Table S1.

AMR genes were identified in the draft genomes using ResFinder (Zankari et al., 2012), with a threshold of 98% identity and minimum length of 60%. Alternatively, the genomes were queried by BLASTn and tBLASTn using sequences of known resistance genes from *A. pleuropneumoniae* and other members of the *Pasteurellaceae* found in GenBank (Supplementary Table S2). Clustal alignments were used to compare the *gyrA, gyrB, parC*, and *parE* genes in isolates with elevated MICs for enrofloxacin (≥0.25 mg/L) with those genes in all remaining isolates in order to identify any mutations that could contribute to enrofloxacin resistance. The profiles of resistance genes identified were then compared to the results of the MIC assays, and where discrepancies were found, isolates were re-tested for susceptibility by disk diffusion, and by PCR for the presence of the specific AMR genes using primers listed in **Table 1**. Correlations between resistance phenotypes and genotypes were calculated using Fisher’s exact test function (‘fisher.test’) in R (version 3.3.2).

**Table 1:** Primers used in this study.

Primer name	Sequence	Amplicon size	Source
sul2_for	TCAACATAACCTCGGACAGTTTCTC	212 bp	Bossé et al., 2015a
sul2_rev	GGGAATGCCATCTGCCTTGAGC		
dfrA14_for	CATTGATAGCTGCGAAAGCGAAAAACGGC	343 bp	Bossé et al., 2015b
dfrA14_rev	ATCGTCGATAAGTGGAGCGTAGAGGC		
blaRob_for	GCTGACATTAACGGCTTGTTCGC	820 bp	This study
blaRob_rev	TTTGGCTTCTTCGGTAAATTGCG		
tetB_for	TTTGCGCTGTAGTGCTCCAAT	944 bp	This study
tetB_rev	AACAAATAAAGTTGCTCGAAAGTA		
tetH_for	TAAATACGGCAGAAAACCCATCTTGC	106 bp	This study
tetH_rev	GCGCCCAATATAGAGCATCCAAAGTG		

## Results

Full results of the testing and identification of AMR genes for each isolate are also shown in Supplementary Table S1. The correlations between presence of identified AMR genes and resistance phenotype are shown in **Table 2**. The majority of the 96 isolates tested in this study, representing serovars 2 (11.5%), 6 (7.3%), 7 (10.4%), 8 (68.8%), and 12 (2.1%), were collected between 2005 and 2009, with smaller numbers representing the other years between 1998 and 2011 (Supplementary Table S1). Only 33% of the isolates did not have any AMR genes, whereas 20% carried a single, and 47% two or more AMR genes. None of the isolates tested were resistant to florefenicol, and no florfenicol resistance genes were detected in the genomes.

**Table 2:** Correlation of phenotypic resistance to selected antimicrobial agents and presence of specific resistance genes detected in draft genomes of 96 *Actinobacillus pleuropneumoniae* isolates from the UK.

Antimicrobialagent^a^	Number of isolatesresistant by genotype^b^	Number of isolates resistant by phenotype (associated MIC)	Correlation of genotype with phenotype^c^
Tetracycline	55	58 (MIC ≥ 4 mg/L)	94.8% (*p* < 2.2e-16)
Ampicillin	19	19 (MIC ≥ 4 mg/L)	100% (*p* < 2.2e-16)
Sulfisoxazole	46	46 (MIC ≥ 256 mg/L, or DD = 6 mm)^d^	100% (*p* < 2.2e-16)
Trimethoprim	16	16 (MIC ≥ 32 mg/L)	100% (*p* < 2.3e-16)
Enrofloxacin	6	6 (MIC ≥ 0.25 mg/L)	100% (*p* < 1.1e-09)

*^a^No isolates were resistant to florefenicol, and no florfenicol resistance genes were detected in the genomes. No specific macrolide resistance genes were identified, and phenotypic resistance to erythromycin, tilmicosin and tylosin was not clear from the MIC values obtained (see Supplementary Table S1 for further details). ^b^Specific genes detected for each resistance phenotype: *tet*(B) or *tet*(H) for tetracycline, *bla*_*ROB-1*_ for ampicillin, *sul2* for sulfisoxazole; *dfrA14* for trimethoprim, and GyrAS83F for enrofloxacin resistant isolates. ^c^Correlation between resistant phenotype and genotype was calculated using Fisher’s exact test function (‘fisher.test’) in R (version 3.3.2). ^d^Initially, two isolates with *sul2* identified in their genomes had an MIC ≤ 16 mg/L, however re-test by disk diffusion showed a zone of inhibition of 6 mm, indicative of resistance. Poor solubility of sulfisoxazole may have caused the discrepancy.*

The MICs for ampicillin showed clear separation into low MIC (MIC ≤ 2 mg/L) for isolates with no resistance gene detected, and high MIC (MIC ≥ 8 mg/L) for 18 isolates with *bla*_ROB-1_ detected by ResFinder. One isolate (MIDG3354) with an MIC = 4 mg/L also had *bla*_ROB-1_ detected by ResFinder. Thus, there was 100% correlation between the presence of *bla*_ROB-1_ and an MIC ≥ 4 mg/L for ampicillin. In all cases, the *bla*_ROB-1_ gene identified showed 99.9% identity with that found in the plasmid pB1000 (accession number DQ840517) from *Haemophilus parasuis* (San Millan et al., 2007). For all but one isolate, the full 918 bp *bla*_ROB-1_ sequence was identified on a single contig. In MIDG3443, where only 857/918 bp were identified by ResFinder, BLASTn confirmed that the entire *bla*_ROB-1_ sequence was present, but split over two contigs.

For tetracycline, 94.8% of isolates considered phenotypically resistant (MIC ≥ 4 mg/L) were found to carry either *tet*(B) or *tet*(H). A 100% correlation was found between the presence either of these genes and an MIC ≥ 8 mg/L. We found of four isolates with an MIC = 4 mg/L, only one (MIDG2567) carried a resistance gene, *tet*(B), whereas three other isolates (MIDG3342, MIDG3352, and MIDG3356) had no detectable tetracycline resistance gene. The *tet*(B) gene identified by ResFinder showed 99.9% to 100% identity with a 1206 bp gene found in the *Shigella flexneri* 2a SRL pathogenicity island (accession number AF326777) or the *S. flexneri* 2b plasmid R100 (accession number AP000342). In some cases, the gene was split over two contigs (with only one part identified by ResFinder), as confirmed by BLASTn. In five isolates, *tet*(H) genes were identified that had either 99.9% identity with the 1179 bp gene found in *Pasteurella aerogenes* plasmid pPAT1 (accession number AJ245947) or 100% identity with the 1203 bp sequence found in *Pasteurella multocida* plasmid pPMT1 (accession number Y15510). These two genes differ at their 3′ ends, and BLASTn analysis confirmed the presence of the 1203 bp gene in four *A. pleuropneumoniae* isolates, and the 1179 bp sequence in one.

For trimethoprim, there was a 100% correlation between an MIC ≥ 32 mg/L and the presence of *dfrA14* in 16 isolates, as previously described (Bossé et al., 2015b). Isolates lacking *dfrA14* had an MIC ≤ 4 mg/L, showing clear separation from the trimethoprim-resistant isolates. There was also a 100% correlation between an MIC ≥ 256 mg/L for sulfisoxazole and the presence of the *sul2* gene in 44 isolates (MIC ≥ 512 mg/L for 39 of these). In two isolates, MIDG2652 and MID3362, initial results (MIC ≤ 16 mg/L) indicated susceptibility despite the presence of the *sul2* gene. PCR confirmed the presence of *sul2* in these isolates, and resistance to sulfisoxazole was confirmed by disk diffusion (zone of inhibition = 6 mm). The *sul2* gene identified by ResFinder shared 100% with an 816 bp gene from *Acinetobacter bereziniae* (accession number GQ421466). In cases where less than 816 bp of the gene were identified by ResFinder, BLASTn confirmed the presence of the remaining sequence on a separate contig.

The six isolates with elevated MICs for enrofloxacin (≥0.25 mg/L) all had identical sequences for their *gyrA, gyrB, parA*, and *parE* genes. The amino acid sequence for each conserved protein encoded by these genes was used to search (by tBLASTn) the remaining genomes in order to determine if any mutations could account for the increased resistance to enrofloxacin. Compared to the sequences found in the enrofloxacin-sensitive isolates, no mutations were found in GyrB, ParA or ParC, however, a substitution in the GyrA sequence (S83F) was found only in the six isolates with MICs for enrofloxacin ≥ 0.25 mg/L.

All isolates had an MIC ≤ 16 mg/L for both erythromycin and tilmicosin, whereas there was a clear separation into low (MIC ≤ 2 mg/L) and high (MIC ≥ 32) levels of resistance to tylosin. However, no macrolide resistance genes were identified by ResFinder in any of the isolates, nor were any mutations detected in the 23S rRNA gene, or in the genes encoding ribosomal proteins L4 and L22 (*rplD* and *rplV*, respectively).

## Discussion

Recently, several groups have investigated the use of genome sequencing as an alternative/adjunct to phenotypic testing for detection and surveillance of AMR in different bacteria (Zankari et al., 2013; Gordon et al., 2014; Köser et al., 2014; Walker et al., 2015; Zhao et al., 2016). For each bacterium, it is necessary to determine the correlation between genetic detection of a resistance gene with phenotypic determination of resistance in order to validate the usefulness of a wgs approach.

In this study, we investigated 96 clinical isolates of *A. pleuropneumoniae*, an important respiratory pathogen of pigs, and found a high correlation between the presence of specific AMR genes and elevated MICs for the corresponding antimicrobial agents tested. However, the results of macrolide testing are inconclusive. Although 20 isolates had an MIC ≥ 32 mg/L for tylosin (seven of which had an MIC ≥ 64 mg/L), all isolates had an MIC ≤ 16 mg/L for both erythromycin and tilmicosin, and no macrolide resistance genes were detected by ResFinder. Furthermore, no point mutations known to confer resistance to macrolides (e.g., in *rplV, rplD, rumA*, or the 23S rRNA genes) were detected by BLASTn in any of the isolates with elevated MICs for tylosin. In other studies, where specific macrolide resistance genes were identified in members of the *Pasteurellaceae* (Olsen et al., 2015; Dayao et al., 2016), higher MIC values (≥64 mg/L) were reported for tilmicosin and erythromycin. There is no established CLSI clinical MIC breakpoint for tylosin resistance in *A. pleuropneumoniae*, and a recent VetPath survey (El Garch et al., 2016) also showed that although only 1/158 *A. pleuropneumoniae* isolates tested had an MIC ≥ 32 mg/L for tilmicosin (considered resistant), 144/158 had an MIC ≥ 32 mg/L for tylosin (none considered resistant due to lack of established breakpoint). From these and our results, it is not clear if there is a specific resistance to tylosin associated with an MIC ≥ 64 mg/L, and if so, what is the mechanism of this resistance.

All six isolates with an MIC ≥ 0.25 mg/L for enrofloxacin had an identical amino acid exchange (S83F) in the GyrA protein, which is in agreement with a previous study in *A. pleuropneumoniae* (Wang et al., 2010). Wang et al. (2010) also reported that combinations of this and other mutations in *gyrA, parC*, and *parE* were associated with higher levels of resistance to enrofloxacin (MIC ≥ 1 mg/L), but these were not detected in our isolates. The six isolates with reduced susceptibility to enrofloxacin were isolated from samples submitted to the same APHA laboratory (Langford), and five were in the same year. Enrofloxacin resistance does not appear to be widespread in *A. pleuropneumoniae* isolates in the UK.

For trimethoprim resistance, there was a 100% correlation between the presence of *dfrA14* and an MIC ≥ 32 mg/L, as previously reported (Bossé et al., 2015b). Although only 16 of the tested isolates were resistant to trimethoprim, all of these also carried the *sul2* gene and had an MIC ≥ 512 mg/L for sulfisoxazole. The *sul2* gene was detected in a total of 46 isolates, however, phenotypic detection of resistance to sulfisoxazole was problematic in some of these. Although 85% of the isolates carrying *sul2* had an MIC ≥ 512 mg/L, and 96% an MIC ≥ 256 mg/L, two isolates initially showed an MIC ≤ 16 mg/L. When re-tested by disk diffusion, these two isolates showed a zone of 6 mm, consistent with a resistant phenotype (Clinical and Laboratory Standards Institute [CLSI], 2013). Issues with the solubility of sulfisoxazole may account for the discrepancies. Co-location of the *dfrA14* and *sul2* genes in two distinct plasmids (Bossé et al., 2015b) accounts for the 16 isolates with resistance to both trimethoprim and sulfisoxazole. Analysis of the sequences flanking *sul2* in the isolates lacking *dfrA14* indicate other possible plasmids that will require further investigation. Only two of the isolates carrying *sul2* had no other detectable AMR genes. The remaining 44 all had *tet*(B) or *tet*(H), with eighteen also carrying *bla*_ROB-1_, and eight of these with *dfrA14* as well. A further eight isolates carried a combination of *tet*(B), *sul2* and *dfrA14*, but lacked the *bla*_ROB-1_ gene (Supplementary Table S1).

There was a 100% correlation between the presence of *bla*_ROB-1_ in 19 isolates and an MIC ≥ 4 mg/L for ampicillin; with an MIC ≥ 8 mg/L in 18 of these. The sequences flanking *bla*_ROB-1_ indicate possible plasmids. Further investigation is required in order to determine if these represent known plasmid(s) described in other *Pasteurellaceae*, such as pB1000 (San Millan et al., 2007) or pJMA-1 (Moleres et al., 2015) or unique plasmids not yet described.

In 98% of isolates carrying either *tet*(B) or *tet*(H), an MIC ≥ 8 mg/L for tetracycline was detected. One isolate with an MIC = 4 mg/L contained the *tet*(B) gene, whereas a further three isolates with an MIC = 4 mg/L did not have any tetracycline resistance genes detected. The reason for this discrepancy is not clear. The sequences flanking *tet*(H) indicate possible plasmid(s), whereas *tet*(B) was found either on small contigs with sequences related to possible plasmid(s), or on larger contigs with sequences indicating a chromosomal insertion site. In 21 isolates with the latter, we recently identified a 56 kb integrative conjugative element (ICE), ICE*Apl1*, inserted in a tRNA-Leu (TAA) gene, which contains *tet*(B) as part of a Tn*10* insertion (Bossé et al., 2016). In 15 other isolates with *tet*(B) on large contigs, the sequence appears to part of a Tn*7* insertion disrupting the competence related gene, *comM* (see Supplementary Table S1). The majority (55/96) of isolates tested were resistant to tetracycline, and only 11 of these carried no other detectable AMR gene.

Reproducibility of phenotypic results has been an issue with surveillance of AMR, with calls for a more standardized method to allow direct comparisons between labs (Michael et al., 2015; El Garch et al., 2016). Our results confirm that wgs is a valuable method that can be used, either in combination with phenotypic testing or on its own, for surveillance of AMR in *A. pleuropneumoniae*. In each isolate where an AMR gene was identified, phenotypic results confirmed resistance. In the case of macrolides, no genes were identified and it is not clear what MIC for tylosin should be considered as a breakpoint indicating resistance. Genome sequencing not only identifies specific AMR genes, but also gives an indication of their locations, either within the chromosome or on plasmids. ResFinder was useful for identifying and locating known AMR gene sequences within the draft genomes, though in cases where a sequence was split over multiple contigs, only the contig with the larger proportion of the gene was identified. ResFinder is also not capable of detecting point mutations in genes such as *gyrA* that lead to a resistance phenotype. In these cases, BLASTn or tBLASTn can be used to either detect mutations or to determine the location of missing sequences not identified by ResFinder.

Plasmids are the biggest contributors to the spread of AMR, with dissemination occurring not only through clonal expansion of the isolates harboring them, but also by conjugal transfer when genes expressing the required machinery are present. Tracking the spread of AMR genes/plasmids provides important epidemiological information that can best be provided by wgs data. Plasmid sequences are often distributed over multiple small contigs within draft genomes, suggesting either repeat sequences within the plasmid, or the presence of multiple plasmids sharing common backbone sequences. Plasmid isolation and sequencing can determine if a single or multiple small plasmids are present, as has been described for *P. multocida* (San Millan et al., 2009), especially in the case of isolates resistant to multiple antimicrobial agents. Most small plasmids that have been characterized in *A. pleuropneumoniae* and other *Pasteurellaceae* are mobilisable, but do not harbor genes encoding conjugal transfer machinery (Blanco et al., 2007; Matter et al., 2008; Kang et al., 2009; San Millan et al., 2009; Bossé et al., 2015a,b). These plasmids can be disseminated either via clonal expansion or conjugation. The latter mechanism requires the presence of other replicons encoding the required machinery, such as the recently described ICE (ICE*Apl1*, ICE*Mh1*, ICE*Pmu1*), discovered in the genomes of different *Pasteurellaceae* species (Michael et al., 2012; Eidam et al., 2015; Bossé et al., 2016). As more wgs become available for members of the *Pasteurellaceae*, it is likely that more ICE will be identified, and their role in dissemination of small plasmids can be further investigated.

In summary, genome sequencing is likely to be used increasingly in the surveillance of AMR, providing a standard method that can be easily compared between different laboratories in different countries. This method can be used on its own for identification of known resistance genes, or in conjunction with sensitivity testing where resistance mechanisms have yet to be identified. Furthermore, wgs data can provide more information than just AMR genotypes, it can also give insights into the mechanisms allowing spread of resistance amongst isolates.

## Members of BRaDP1T Consortium

The BRaDP1T Consortium comprises: Duncan J. Maskell, Alexander W. (Dan) Tucker, Sarah E. Peters, Lucy A. Weinert, Jinhong (Tracy) Wang, Shi-Lu Luan, Roy R. Chaudhuri (University of Cambridge), Andrew N. Rycroft, Gareth A. Maglennon, Jessica Beddow (Royal Veterinary College); Brendan W. Wren, Jon Cuccui, Vanessa S. Terra (London School of Hygiene and Tropical Medicine); and Paul R. Langford, Janine T. Bossé, Yanwen Li (Imperial College London).

## Author Contributions

JB, PL, AR, BW, DM, AT conceived the study; JB, YaL, JR, RF, YiL, RRC, and MH produced the data; JB, YaL, RF, RRC analyzed the data; JB, PL wrote the paper.

## Conflict of Interest Statement

The authors declare that the research was conducted in the absence of any commercial or financial relationships that could be construed as a potential conflict of interest.

## Footnotes

1 https://github.com/najoshi/sickle

2 http://www.ebi.ac.uk/ena
