# Supplementary material for: Whole Genome Sequencing for Surveillance of Antimicrobial Resistance in Actinobacillus pleuropneumoniae
Source: Front Microbiol. 2017 Mar 6;8:311. doi: 10.3389/fmicb.2017.00311 (PMC5337627; doi:10.3389/fmicb.2017.00311)
Supplement: Supplementary file 1 [file Table_1.docx]

Table S1. Phenotypic and genotypic analysis of antimicrobial resistance (AMR) in clinical isolates of *A. pleuropneumoniae*.

|  |  |  |  | Tetracylcline | | Ampicilin | | Sulfisoxazole | | Trimethoprim | | Enrofloxacin | | Tilmicosin | Tylosin | Erythromycin | Accession number |
| --- | --- | --- | --- | --- | --- | --- | --- | --- | --- | --- | --- | --- | --- | --- | --- | --- | --- |
| Isolate ID | Serovar | Location | Year | MIC | Gene | MIC | Gene | MIC | Gene | MIC | Gene | MIC | Gene | MIC | MIC | MIC |  |
| MIDG2249 | 7 | B St E | 1998 | 1 |  | 1 |  | 32 |  | ≤0.25 |  | 0.032 |  | 16 | 2 | 16 | ERS134606 |
| MIDG2250 | 7 | B St E^a^ | 1998 | 1 |  | 1 |  | 32 |  | ≤0.25 |  | 0.032 |  | 16 | 2 | 16 | ERS134309 |
| MIDG2356 | 7 | B St E | 1998 | **16** | *tet*(B )^#^ | **≥8** | *bla*_ROB-1_ | **≥512** | *sul2* | **>32** | *dfrA14* | 0.032 |  | 16 | 32 | 16 | ERS134310 |
| MIDG2465 | 7 | B St E | 1999 | 1 |  | 1 |  | 16 |  | ≤0.25 |  | 0.032 |  | 16 | 2 | 16 | ERS134607 |
| MIDG2537 | 7 | B St E | 2002 | 1 |  | 1 |  | 16 |  | ≤0.25 |  | 0.032 |  | 16 | 2 | 16 | ERS134312 |
| MIDG2567 | 8 | Thirsk | 2003 | 4 | *tet*(B) | 1 |  | **≥512** | *sul2* | 0.5 |  | 0.016 |  | 4 | 2 | 8 | ERS134321 |
| MIDG2615 | 7 | B St E | 2004 | 1 |  | 1 |  | 32 |  | ≤0.25 |  | 0.032 |  | 16 | 2 | 16 | ERS134313 |
| MIDG2620 | 8 | Preston | 2004 | **16** | *tet*(B)^#^ | 1 |  | **≥512** | *sul2* | ≤0.25 |  | 0.032 |  | 8 | 2 | 16 | ERS134706 |
| MIDG2648 | 8 | B St E | 2005 | **8** | *tet*(B)*** | 1 |  | **≥512** | *sul2* | 0.5 |  | 0.032 |  | 8 | 2 | 8 | ERS134315 |
| MIDG2650 | 8 | Thirsk | 2005 | **16** | *tet*(B) | 1 |  | **≥512** | *sul2* | ≤0.25 |  | 0.032 |  | 16 | 2 | 16 | ERS134316 |
| MIDG2651 | 2 | Thirsk | 2005 | 1 |  | 1 |  | <256 |  | 2 |  | 0.032 |  | 8 | 2 | 16 | ERS134609 |
| MIDG2652 | 8 | Thirsk | 2005 | **8** | *tet*(B)*** | 2 |  | **6=R DD**^b^ | *sul2* | ≤0.25 |  | 0.032 |  | 8 | n/d | 16 | ERS134318 |
| MIDG2653 | 2 | Thirsk | 2005 | 1 |  | 1 |  | 128 |  | ≤0.25 |  | 0.032 |  | 16 | 2 | 16 | ERS134319 |
| MIDG2656 | 2 | Winchester | 2005 | **16** | *tet*(B) | 1 |  | 64 |  | n/d^c^ |  | 0.032 |  | 16 | 2 | 16 | ERS134610 |
| MIDG2657 | 8 | Winchester | 2005 | **8** | *tet*(B)*** | 1 |  | **≥512** | *sul2* | **>32** | *dfrA14* | 0.016 |  | 8 | 2 | 16 | ERS134321 |
| MIDG2658 | 8 | Thirsk | 2005 | **16** | *tet*(B) | 1 |  | **≥512** | *sul2* | 2 |  | 0.032 |  | 8 | 2 | 8 | ERS134322 |
| MIDG2659 | 8 | B St E | 2005 | **8** | *tet*(B)^#^ | **≥8** | *bla*_ROB-1_ | **≥512** | *sul2* | 2 |  | 0.032 |  | 16 | 64 | 16 | ERS134771 |
| MIDG2660 | 8 | B St E | 2005 | 1 |  | 1 |  | 16 |  | ≤0.25 |  | n/d |  | 8 | 2 | 8 | ERS134324 |
| MIDG2661 | 8 | B St E | 2005 | **16** | *tet*(B) | 1 |  | <256 |  | ≤0.25 |  | 0.032 |  | 8 | 2 | 8 | ERS134325 |
| MIDG2662 | 2 | B St E | 2005 | 1 |  | 2 |  | 32 |  | ≤0.25 |  | 0.032 |  | 16 | 2 | 16 | ERS134612 |
| MIDG2663 | 8 | Thirsk | 2005 | **8** | *tet*(B)*** | 1 |  | **≥512** | *sul2* | ≤0.25 |  | 0.032 |  | 8 | 2 | 16 | ERS134327 |
| MIDG2664 | 8 | B St E | 2005 | **8** | *tet*(B)*** | 1 |  | **≥512** | *sul2* | **>32** | *dfrA14* | 0.032 |  | 8 | 2 | 16 | ERS134328 |
| MIDG2665 | 8 | B St E | 2005 | 1 |  | 1 |  | 32 |  | 0.5 |  | 0.032 |  | 8 | 2 | 16 | ERS134613 |
| MIDG2666 | 8 | B St E | 2005 | **>16** | *tet*(B) | 1 |  | **≥512** | *sul2* | 0.5 |  | 0.016 |  | 8 | 2 | 8 | ERS134330 |
| MIDG3200 | 8 | Thirsk | 2006 | **8** | *tet*(B)*** | 1 |  | **≥512** | *sul2* | ≤0.25 |  | 0.032 |  | 16 | 2 | 16 | ERS134331 |
| MIDG3201 | 8 | B St E | 2006 | **8** | *tet*(B)*** | 1 |  | **≥512** | *sul2* | **>32** | *dfrA14* | 0.016 |  | 8 | 2 | 16 | ERS134708 |
| MIDG3202 | 8 | B St E | 2006 | **16** | *tet*(B) | 1 |  | 16 |  | ≤0.25 |  | 0.032 |  | 8 | 2 | 16 | ERS134333 |
| MIDG3203 | 6 | B St E | 2006 | 1 |  | 1 |  | <256 |  | 4 |  | 0.12 |  | 16 | 2 | 16 | ERS134334 |
| MIDG3204 | 6 | B St E | 2006 | 1 |  | 1 |  | <256 |  | ≤0.25 |  | 0.032 |  | 16 | 2 | 16 | ERS134615 |
| MIDG3205 | 8 | Shrewsbury | 2006 | 1 |  | 1 |  | 32 |  | ≤0.25 |  | 0.032 |  | 8 | 2 | 16 | ERS134336 |
| MIDG3206 | 6 | Thirsk | 2006 | 2 |  | 1 |  | 16 |  | ≤0.25 |  | 0.032 |  | 8 | 2 | 16 | ERS134337 |
| MIDG3207 | 8 | Thirsk | 2006 | 1 |  | 1 |  | 16 |  | ≤0.25 |  | 0.032 |  | 8 | 2 | 16 | ERS134616 |
| MIDG3208 | 8 | B St E | 2006 | 1 |  | 1 |  | 8 |  | ≤0.25 |  | 0.016 |  | 8 | 1 | 16 | ERS134339 |
| MIDG3209 | 2 | Thirsk | 2006 | 1 |  | 1 |  | 32 |  | ≤0.25 |  | 0.032 |  | 8 | 2 | 16 | ERS134340 |
| MIDG3211 | 8 | Preston | 2006 | **16** | *tet*(B)^#^ | 1 |  | **≥512** | *sul2* | 0.5 |  | 0.032 |  | 8 | 2 | 16 | ERS134342 |
| MIDG3212 | 8 | Preston | 2006 | **16** | *tet*(B)^#^ | 2 |  | **≥512** | *sul2* | 1 |  | 0.032 |  | 8 | 2 | 16 | ERS134343 |
| MIDG3213 | 8 | Langford | 2006 | 1 |  | 1 |  | 32 |  | ≤0.25 |  | **0.25** | *gyrA*S83F | 8 | 2 | 16 | ERS134618 |
| MIDG3214 | 8 | Langford | 2006 | 1 |  | 1 |  | 32 |  | ≤0.25 |  | **0.25** | *gyrA*S83F | 8 | 2 | 16 | ERS134345 |
| MIDG3216 | 8 | Langford | 2006 | 1 |  | 1 |  | 32 |  | ≤0.25 |  | **0.25** | *gyrA*S83F | 8 | 2 | 16 | ERS134619 |
| MIDG3217 | 8 | Langford | 2006 | 1 |  | 1 |  | 32 |  | ≤0.25 |  | **0.25** | *gyrA*S83F | 8 | 2 | 16 | ERS134348 |
| MIDG3220 | 8 | Langford | 2006 | 1 |  | 1 |  | 32 |  | ≤0.25 |  | **0.25** | *gyrA*S83F | 8 | 2 | 8 | ERS134351 |
| MIDG3221 | 8 | Langford | 2006 | **8** | *tet*(B)*** | 1 |  | **>256** | *sul2* | **>32** | *dfrA14* | 0.016 |  | 8 | 2 | 16 | ERS134352 |
| MIDG3223 | 8 | Thirsk | 2007 | 1 |  | 1 |  | 16 |  | ≤0.25 |  | 0.032 |  | 8 | 2 | 16 | ERS134354 |
| MIDG3224 | 8 | B St E | 2007 | 1 |  | 1 |  | **≥512** | *sul2* | **>32** | *dfrA14* | 0.032 |  | 8 | 2 | 16 | ERS134355 |
| MIDG3226 | 8 | B St E | 2007 | **8** | *tet*(B)^#^ | **≥8** | *bla*_ROB-1_ | **≥512** | *sul2* | 2 |  | 0.032 |  | 8 | 64 | 8 | ERS134357 |
| MIDG3227 | 6 | Langford | 2007 | 1 |  | 1 |  | 32 |  | ≤0.25 |  | 0.016 |  | 16 | 2 | 16 | ERS134358 |
| MIDG3228 | 8 | Thirsk | 2007 | 2 |  | 1 |  | 32 |  | ≤0.25 |  | 0.016 |  | 8 | 2 | 8 | ERS134711 |
| MIDG3229 | 8 | Thirsk | 2007 | **8** | *tet*(B)*** | 2 |  | **≥512** | *sul2* | ≤0.25 |  | 0.016 |  | 8 | 2 | 16 | ERS134360 |
| MIDG3230 | 8 | Thirsk | 2007 | **16** | *tet*(B)^#^ | 2 |  | **≥512** | *sul2* | 1 |  | 0.032 |  | 8 | 2 | 8 | ERS134361 |
| MIDG3232 | 8 | Thirsk | 2007 | **8** | *tet*(B)*** | **>8** | *bla*_ROB-1_ | **≥512** | *sul2* | **>32** | *dfrA14* | 0.016 |  | 8 | 64 | 8 | ERS134363 |
| MIDG3233 | 6 | Langford | 2008 | **8** | *tet*(B) | 1 |  | 16 |  | ≤0.25 |  | 0.032 |  | 16 | 2 | 16 | ERS134364 |
| MIDG3234 | 8 | Starcross | 2008 | **16** | *tet*(B) | 2 |  | 16 |  | ≤0.25 |  | 0.032 |  | 8 | 2 | 16 | ERS134625 |
| MIDG3341 | 8 | B St E | 2004 | **16** | *tet*(B)^#^ | 1 |  | **≥512** | *sul2* | 0.5 |  | 0.032 |  | 8 | 2 | 16 | ERS134367 |
| MIDG3342 | 2 | B St E | 2005 | 4 |  | 1 |  | 16 |  | n/d |  | 0.016 |  | 8 | 2 | 16 | ERS134712 |
| MIDG3344 | 8 | Langford | 2005 | **8** | *tet*(B)*** | 1 |  | 16 |  | ≤0.25 |  | 0.032 |  | 8 | 2 | 16 | ERS134369 |
| MIDG3345 | 8 | Preston | 2005 | **16** | *tet*(B)^#^ | 1 |  | **≥512** | *sul2* | 1 |  | 0.032 |  | 8 | 2 | 16 | ERS134370 |
| MIDG3346 | 8 | Thirsk | 2005 | **8** | *tet*(B)*** | **≥8** | *bla*_ROB-1_ | **≥512** | *sul2* | **>32** | *dfrA14* | 0.016 |  | 8 | 64 | 16 | ERS134627 |
| MIDG3347 | 12 | Langford | 2006 | 1 |  | 1 |  | 16 |  | ≤0.25 |  | 0.032 |  | 16 | 2 | 16 | ERS134372 |
| MIDG3349 | 8 | Thirsk | 2006 | **8** | *tet*(B)*** | 1 |  | **≥512** | *sul2* | **>32** | *dfrA14* | 0.032 |  | 8 | 2 | 16 | ERS134628 |
| MIDG3351 | 8 | Langford | 2006 | 1 |  | 1 |  | 32 |  | ≤0.25 |  | **0.250** | *gyrA*S83F | 8 | 2 | 16 | ERS134376 |
| MIDG3352 | 8 | Thirsk | 2006 | 4 |  | 1 |  | **≥512** | *sul2* | 2 |  | 0.032 |  | 8 | 2 | 16 | ERS134773 |
| MIDG3354 | 2 | Thirsk | 2007 | **8** | *tetH* | **4** | *bla*_ROB-1_ | 32 |  | ≤0.25 |  | 0.016 |  | 16 | 32 | 16 | ERS134378 |
| MIDG3356 | 8 | B St E | 2007 | 4 |  | 0.5 |  | **≥512** | *sul2* | 0.5 |  | 0.016 |  | 8 | 1 | 8 | ERS134379 |
| MIDG3358 | 6 | B St E | 2008 | n/d |  | 1 |  | 32 |  | 1 |  | n/d |  | n/d | 2 | n/d | ERS134630 |
| MIDG3360 | 8 | Thirsk | 2008 | **16** | *tetH* | **≥8** | *bla*_ROB-1_ | **≥512** | *sul2* | 0.5 |  | 0.032 |  | 8 | 64 | 8 | ERS134381 |
| MIDG3362 | 12 | Thirsk | 2008 | **8** | *tet*(B) | **>16** | *bla*_ROB-1_ | **6=R DD** | *sul2* | ≤0.25 |  | 0.032 |  | 16 | 2 | 16 | ERS134382 |
| MIDG3364 | 2 | Thirsk | 2008 | 1 |  | 0.5 |  | 8 |  | ≤0.25 |  | 0.016 |  | 16 | 1 | 16 | ERS134384 |
| MIDG3366 | 2 | B St E | 2008 | 1 |  | 0.5 |  | 8 |  | ≤0.25 |  | 0.016 |  | 8 | 1 | 16 | ERS134385 |
| MIDG3367 | 2 | B St E | 2008 | 1 |  | 0.5 |  | 8 |  | ≤0.25 |  | 0.016 |  | 8 | 1 | 8 | ERS134714 |
| MIDG3368 | 8 | Thirsk | 2008 | **8** | *tet*(B)*** | 1 |  | **≥512** | *sul2* | 2 |  | 0.016 |  | 8 | 2 | 16 | ERS134387 |
| MIDG3369 | 8 | B St E | 2008 | **8** | *tet*(B)^#^ | **≥8** | *bla*_ROB-1_ | **≥512** | *sul2* | n/d |  | 0.032 |  | 8 | >64 | 16 | ERS134388 |
| MIDG3370 | 8 | Thirsk | 2009 | **8** | *tet*(B)*** | **≥8** | *bla*_ROB-1_ | **≥512** | *sul2* | **>32** | *dfrA14* | 0.032 |  | 8 | 32 | 8 | ERS134633 |
| MIDG3371 | 8 | Thirsk | 2009 | **8** | *tet*(B)*** | **≥8** | *bla*_ROB-1_ | **≥512** | *sul2* | **>32** | *dfrA14* | 0.032 |  | 8 | 32 | 8 | ERS134390 |
| MIDG3372 | 8 | Thirsk | 2009 | **8** | *tet*(B)*** | **≥8** | *bla*_ROB-1_ | **≥512** | *sul2* | **>32** | *dfrA14* | 0.032 |  | 8 | 32 | 8 | ERS134391 |
| MIDG3374 | 2 | B St E | 2009 | n/d |  | 1 |  | 8 |  | n/d |  | 0.032 |  | n/d | 1 | n/d | ERS134634 |
| MIDG3376 | 6 | Shrewsbury | 2008 | 1 |  | 2 |  | 8 |  | ≤0.25 |  | 0.064 |  | 16 | 2 | 16 | ERS134394 |
| MIDG3377 | 8 | B St E | 2009 | **8** | *tetH* | 2 |  | 32 |  | ≤0.25 |  | 0.032 |  | 8 | 2 | 8 | ERS134715 |
| MIDG3378 | 8 | B St E | 2009 | **8** | *tet*(B)*** | 1 |  | **≥512** | *sul2* | **>32** | *dfrA14* | 0.016 |  | 8 | 2 | 8 | ERS134396 |
| MIDG3379 | 8 | Thirsk | 2009 | **16** | *tet*(B) | 1 |  | **≥512** | *sul2* | 1 |  | 0.016 |  | 8 | 2 | 8 | ERS134397 |
| MIDG3380 | 8 | Thirsk | 2009 | **16** | *tet*(B) | 1 |  | **≥512** | *sul2* | 1 |  | 0.032 |  | 8 | 2 | 8 | ERS134636 |
| MIDG3382 | 8 | Thirsk | 2009 | **8** | *tet*(B) | 1 |  | 8 |  | ≤0.25 |  | 0.032 |  | 16 | 2 | 16 | ERS134400 |
| MIDG3383 | 8 | B St E | 2009 | **8** | *tet*(B)^#^ | **≥8** | *bla*_ROB-1_ | **≥512** | *sul2* | 2 |  | 0.032 |  | 8 | >64 | 16 | ERS134637 |
| MIDG3384 | 8 | Thirsk | 2009 | 2 |  | 1 |  | 16 |  | ≤0.25 |  | 0.016 |  | 8 | 2 | 16 | ERS134402 |
| MIDG3385 | 8 | Thirsk | 2009 | 1 |  | 1 |  | 16 |  | ≤0.25 |  | 0.016 |  | 8 | 2 | 16 | ERS134403 |
| MIDG3388 | 8 | Thirsk | 2009 | **8** | *tet*(B)*** | **≥8** | *bla*_ROB-1_ | **≥512** | *sul2* | **>32** | *dfrA14* | 0.016 |  | 8 | 32 | 8 | ERS155332 |
| MIDG3389 | 8 | Thirsk | 2009 | **8** | *tet*(B)*** | **≥8** | *bla*_ROB-1_ | **≥512** | *sul2* | **>32** | *dfrA14* | 0.016 |  | 8 | 32 | 16 | ERS155333 |
| MIDG3394 | 7 | Thirsk | 2010 | **8** | *tet*(B) | 1 |  | 16 |  | ≤0.25 |  | 0.032 |  | 16 | 2 | 8 | ERS155334 |
| MIDG3395 | 8 | Thirsk | 2010 | **8** | *tet*(B)*** | 1 |  | **≥512** | *sul2* | **>32** | *dfrA14* | 0.016 |  | 8 | 2 | 8 | ERS155335 |
| MIDG3396 | 8 | Thirsk | 2010 | 2 |  | 1 |  | 16 |  | ≤0.25 |  | 0.016 |  | 8 | 2 | 16 | ERS155336 |
| MIDG3397 | 7 | Thirsk | 2010 | 1 |  | 1 |  | 32 |  | ≤0.25 |  | 0.016 |  | 8 | 2 | 16 | ERS155337 |
| MIDG3443 | 8 | Thirsk | 2011 | **8** | *tet*(B)^#^ | **≥16** | *bla*_ROB-1_ | **>256** | *sul2* | ≤2 |  | ≤0.12 |  | 8 | >32 | n/d | ERS155388 |
| MIDG3444 | 8 | B St E | 2011 | **8** | *tet*(B)^#^ | **≥16** | *bla*_ROB-1_ | **>256** | *sul2* | ≤2 |  | ≤0.12 |  | ≤ 4 | >32 | n/d | ERS155389 |
| MIDG3445 | 8 | B St E | 2011 | **8** | *tet*(B)^#^ | **≥16** | *bla*_ROB-1_ | **>256** | *sul2* | ≤2 |  | ≤0.12 |  | ≤ 4 | >32 | n/d | ERS155390 |
| MIDG3447 | 8 | B St E | 2010 | **8** | *tet*(B)^#^ | **≥16** | *bla*_ROB-1_ | **>256** | *sul2* | ≤2 |  | ≤0.12 |  | 8 | 32 | n/d | ERS155392 |
| MIDG3448 | 7 | Thirsk | 2010 | **8** | *tetH* | ≤ 0.25 |  | <256 |  | ≤2 |  | ≤0.12 |  | 8 | >32 | n/d | ERS155393 |
| MIDG3449 | 7 | B St E | 2011 | **8** | *tetH* | ≤ 0.25 |  | <256 |  | ≤2 |  | ≤0.12 |  | 8 | >32 | n/d | ERS155394 |

MIC values shown in bold text indicate resistance;

^a^B St E = Bury St Edmunds;^#^ in these isolates, the *tet*(B)gene was located in the chromosome as part of a Tn*7* insertion in *comM*;*in these isolates, the *tet*(B)gene was located in the chromosome as part of the 56 kb integrative conjugative element ICE*Apl1* (Bossé et al., 2016).

^b^Initially, two isolates (MIDG2652 and MIDG3362) with *sul2* identified in their genomes had an MIC≤16 mg/L, however re-test by disc diffusion showed a zone of inhibition of 6 mm, indicative of resistance (6=R DD). Poor solubility of sulfisoxazole may have caused the discrepancy.

^c^For some isolates, it was not possible to get results of sensitivity testing for certain antimicrobial agents due to inconsistent growth at the different dilutions. In all cases where this occurred, no AMR genes were detected for the respective antimicrobial agent.
