# Supplementary material for: Whole Genome Sequencing for Surveillance of Antimicrobial Resistance in Actinobacillus pleuropneumoniae
Source: Front Microbiol. 2017 Mar 6;8:311. doi: 10.3389/fmicb.2017.00311 (PMC5337627; doi:10.3389/fmicb.2017.00311)
Supplement: Supplementary file 2 [file Table_2.DOCX]

Table S2. Genbank accession numbers of antimicrobial resistance genes from the *Pasteurellaceae* used to query the *A. pleuropneumoniae* genomes by Blastn.

| Resistance phenotype | Gene | Accession number |
| --- | --- | --- |
| Ampicillin | *bla*_ROB-1_ | ACD76088 |
|  | *bla*_TEM-1_ | AFJ32818 |
|  | *bla*_TEM-15_ | CAO98721 |
|  | *bla*_PSE-1_ | AY232671 |
|  |  |  |
| Tetracycline | *tet*(B) | ANC67579 |
|  | *tet*(H) | YP_001966263 |
|  | *tet*(O) | AAY54279 |
|  | *tet*(M) | BAV93039 |
|  | *tet*(G) | AY232670 |
|  | *tet*(L) | AY359464 |
|  |  |  |
| Sulfisoxazole | *sul2* | YP_245431 |
|  |  |  |
| Trimethoprim | *dfrA14* | KP197004 |
|  |  |  |
| Florfenicol | *floR* | KP696484 |
|  |  |  |
| Enrofloxacin | *gyrA* | ABN73394 |
|  | *gyrB* | ABN73917 |
|  | *parC* | ABN73680 |
|  | *parE* | ABN74341 |
|  |  |  |
| Macrolides | *macA* | ABN73495 |
|  | *macB* | ABN73728 |
|  | *rplV* | ABN74849 |
|  | *rplD* | ABN74845 |
|  | *rumA* | ABN73416 |
|  | *rumB* | ABN74204 |
|  | 23S rRNA | NR_076484 |
